# A gut response

**DOI:** 10.7554/eLife.28152

**Published:** 2017-06-02

**Authors:** Matthew L Nicotra

**Affiliations:** 1 Thomas E. Starzl Transplant Institute and the Departments of Surgery and Immunology, University of Pittsburgh, Pittsburgh, United Statesnicotraml@upmc.edu

**Keywords:** Strongylocentrotus purpuratus, gut immunity, echinoderm, cytokines, Other

## Abstract

Unexpected findings from the immune system of sea urchin larvae potentially provide insights into immune signaling in ancestral animals.

**Related research article** Buckley KM, Ho ECH, Hibino T, Schrankel CS, Schuh NW, Wang G, Rast JP. 2017. IL17 factors are early regulators in the gut epithelium during inflammatory response to *Vibrio* in the sea urchin larva. *eLife* **6**:e23481. doi: 10.7554/eLife.23481

In 1882, Elie Metchnikoff peered through a microscope at a starfish larva and observed migrating cells engulf a splinter. He named these cells ‘phagocytes’, and they inspired the idea that certain cells defend the organism by eating foreign invaders (Metchnikoff, 1893). Metchnikoff’s phagocytosis theory described a new dimension of immunity and earned him a Nobel prize in 1908 (Tauber, 2003).

Starfish are echinoderms – a set of marine animals that also includes sea urchins. Nearly 100 years after Metchnikoff identified phagocytes, a team of scientists sequenced the urchin genome and made a startling discovery: several families of innate immune molecules had more than 200 members, which was much greater than anything encountered in previously sequenced animal genomes (Sea Urchin Genome Sequencing Consortium et al., 2006). As the genomes of additional species were sequenced, a picture emerged indicating that innate immune systems of echinoderms and other invertebrates rely on a genomic complexity not seen in mammals.

Encoded in the genome of many animals are molecules called cytokines that play important signaling roles in the immune system. Now, in eLife, Jonathan Rast, Katherine Buckley and co-workers at the University of Toronto and Sunnybrook Research Institute – including Buckley and Eric Ho as joint first authors – report unexpected results regarding a cytokine called IL-17 in echinoderm larvae (Buckley et al., 2017).

Immunologists focus a lot of attention on how cytokines control immune cells, but they (the cytokines, not the immunologists) can also work on other cell types. For example, in mice and humans, IL-17 acts almost entirely on the epithelial cells that line body cavities and the mesenchymal cells that make up connective tissue. In response to IL-17, the cells trigger inflammation – an important part of the early immune response (Amatya et al., 2017).

While Buckley et al. did not set out to study IL-17, they were interested in how urchins deal with bacteria in their guts. Urchin larvae feed by filtering microbe-rich seawater, and should therefore have robust gut immune responses. The larvae are transparent, which allowed Buckley et al. to watch as the same cell types that enthralled Metchnikoff engulfed bacteria in the gut. Yet even before the infection was visible, immune cells migrated to the gut and the gut epithelium thickened and closed off. This indicated an early, systemic immune response. Further investigation revealed that the urchin versions of IL-17 were some of the most highly expressed genes immediately after an infection. This raised the question of whether IL-17 has an ancient role as a regulator of gut microbial responses.

For most cytokines it would be difficult, if not impossible, to find the answer. Cytokines evolve so quickly that it is hard to identify which are homologs (i.e. which genes share a common ancestor). IL-17, however, has several highly conserved motifs that allowed Buckley et al. to identify 35 members of the IL-17 family in urchins. By contrast, mice and humans only have six members (Amatya et al., 2017). These 35 cytokines cluster into ten subgroups, dubbed SpIL17-1 through SpIL17-10. Remarkably, the subgroups appear in four other echinoderm species that span over 260 million years of evolution. This conservation suggests each family has a distinct and essential role. Unfortunately, more than 500 million years of evolution separates echinoderms and mammals, which prevents us from establishing the evolutionary relationships between individual urchin and mammalian genes, even for IL-17.

Buckley et al. next assembled a detailed picture of IL-17 expression in both adult urchins and larvae. All IL-17s were completely inactive in healthy larvae, but four hours after an infection the activity of two families – SpIL17-1 and SpIL17-4 – dramatically increased. Subsequently, several other immune genes were activated, including two hallmarks of inflammation. A series of cellular localization experiments further demonstrated that gut epithelial cells – and not immune cells – express IL-17 cytokines. Buckley et al. then combined publicly available data with their own experiments to show that in adult urchins, SpIL17-1 and SpIL17-4 are inactive during bacterial infections. Instead, a different family (SpIL17-9) increases its activity, which is followed by the expression of several echinoderm-specific defense molecules.

With 35 IL-17-like cytokines in the genome, one might expect a similar diversity of IL-17 receptors. However, Buckley et al. found only two. Blocking the translation of one of the receptor types was lethal for the larvae. Blocking the other receptor – termed IL-17R1 – reduced the expression of several (but not all) immune response genes, and did not prevent immune cell migration. Other pathways of immune activation must therefore exist.

These findings raise a number of questions. Why do echinoderms have ten IL-17 families yet apparently only two receptors? Do additional IL-17 receptors that lack homology to their vertebrate counterparts exist? How does the gut epithelium sense bacteria to trigger an immune response? One can expect that recent demonstrations of CRISPR/Cas9 mediated genome editing in urchins will help researchers to seek answers to these questions (Lin and Su, 2016).

More fundamentally, does the production of IL-17 by epithelial cells represent an ancestral immune state in animals? The answer could be yes. Nearly everything we know about mammalian IL-17 signaling comes from studies of a subfamily called IL-17A, which is secreted by immune cells, but several other IL-17s are produced by gut epithelial cells (Song et al., 2011).

In summary, these results reinforce the idea that we should study host immune defenses across the animal tree in order to discover general properties of immune systems (Litman and Cooper, 2007). Buckley et al. have achieved this by peering into an echinoderm. Metchnikoff would approve.
